# Parenteral Nutrition and Lipids

**DOI:** 10.3390/nu9040388

**Published:** 2017-04-14

**Authors:** Maitreyi Raman, Abdulelah Almutairdi, Leanne Mulesa, Cathy Alberda, Colleen Beattie, Leah Gramlich

**Affiliations:** 1 Faculty of Medicine, University of Calgary, G055 3330 Hospital Drive, Calgary, AB T2N 4N1, Canada; mut_aaa@yahoo.com; 2 Alberta Health Services, Seventh Street Plaza, 14th Floor, North Tower, 10030–107 Street NW, Edmonton, AB T5J 3E4, Canada; Leanne.Mulesa@albertahealthservices.ca (L.M.); Cathy.Alberda@albertahealthservices.ca (C.A.); Colleen.Beattie@albertahealthservices.ca (C.B.); 3 Division of Gastroenterology, Royal Alexandra Hospital, University of Alberta, Edmonton, AB T5H 3V9, Canada; lg3@ualberta.ca

**Keywords:** parenteral nutrition, lipids, intravenous Lipid Emulsions

## Abstract

Lipids have multiple physiological roles that are biologically vital. Soybean oil lipid emulsions have been the mainstay of parenteral nutrition lipid formulations for decades in North America. Utilizing intravenous lipid emulsions in parenteral nutrition has minimized the dependence on dextrose as a major source of nonprotein calories and prevents the clinical consequences of essential fatty acid deficiency. Emerging literature has indicated that there are benefits to utilizing alternative lipids such as olive/soy-based formulations, and combination lipids such as soy/MCT/olive/fish oil, compared with soybean based lipids, as they have less inflammatory properties, are immune modulating, have higher antioxidant content, decrease risk of cholestasis, and improve clinical outcomes in certain subgroups of patients. The objective of this article is to review the history of IVLE, their composition, the different generations of widely available IVLE, the variables to consider when selecting lipids, and the complications of IVLE and how to minimize them.

## 1. Background of Intravenous Lipid Emulsions (IVLEs)

Intravenous lipid emulsions (IVLEs) are a source of essential fatty acids (EFAs) and energy-dense non-protein calories. In addition, lipids have multiple physiological roles in the body that are biologically vital. Fatty acids form the major constituent of cellular bio-membranes and contribute to membrane integrity, regulate permeability and are precursors to key modulators involved in cellular pathways of the immune system [1]. Furthermore, lipids serve as substrate for de novo biosynthesis of cholesterol and endogenous steroids and as precursors of modulators of inflammation and platelet function (leukotrienes, thromoboxanes) [1]. Lipids consist of triglycerides (TGs), sterols and phospholipids. TGs constitute molecules of glycerol to which three fatty acids (FAs) have been esterified. IVLEs are suspensions of oil in an aqueous medium manufactured to possess properties to naturally occurring chylomicrons, and are unstable systems that are subject to physical changes over time [2]. Although history would expound experimentation with fat administration as early as the 17th century, a safe and viable lipid emulsion was only developed for routine administration in the early 1960s [2]. A soybean oil lipid emulsion (SOY) has been the mainstay of parenteral nutrition lipid formulations since its adoption in Europe with approval in 1961 and subsequently in the United States a decade later.

FAs are an important component of IVLE, and are classified based on several characteristics including the carbon chain length, degree of unsaturation, and location of the first double bond. Short chain FAs (SCFAs) have 2–4 carbons, medium chain FAs (MCFAs) have 6–12 carbons, while long chain FAs (LCFAs) have more than or equal to 14 carbons. Saturated FAs have no double bonds, monounsaturated FAs (MUFAs) have one double bond, and polyunsaturated FAs (PUFAs) have two or more double bonds. The location of the first double bond, counting from the methyl end of the molecule, is called the ω carbon. The fatty acid that has its first double bond occurring in the ninth carbon atom, for example, is called ω-9 FA. In contrast, if the first double bond that occurs in the sixth carbon atom it is called ω-6 FA, and the FA is called ω-3, if the double bond occurs in the third carbon atom [2,3]. Saturated fats can be sub-classified into short chain, medium chain, and long chain fats whereas mono- and polyunsaturated fats are all long chain fats [4].

Fats given intravenously bypass the intestinal lumen and do not undergo hydrolysis by pancreatic lipases, emulsification by bile, or packaging into chylomicrons like dietary fats. Therefore, IV lipids should be prepackaged in a way to enable them being transferred into the hydrophilic physiologic environment. IVLE are prepared structurally similar to chylomicrons using phospholipids emulsifiers coating a TG core. In addition to the phospholipid emulsifier, sodium oleate is added as a stabilizing agent and glycerin is added as an osmotic agent [1].

The TG core of the lipid emulsion serves together with the phospholipid emulsifier as a major source of non-protein calories and supplier of EFA in parenteral nutrition (PN)–dependent patients. TGs are provided from sources such as soybean oil (SOY), olive oil (OO), safflower oil, coconut oil, and fish oil (FO). Each TG source differs with respect to FA content, EFAD potential, and phytosterol and alpha-tocopherol content (see Table 1). Phytosterols are plant-derived sterols that resemble cholesterol structurally but not metabolized by human body. When administered intravenously in animal models, phytosterols may inhibit bile flow resulting in liver cholestasis [5,6]. Alpha-tocopherol serves as anti-oxidant to scavenge free radicals from peroxidized lipids to prevent propagation of oxidative lipid damage [1].

Since the introduction of intravenous lipid emulsions (IVLEs) to the clinical practice, substantial research efforts have continued to make these products stable and safe enough to meet the nutritional demand of patients in requiring PN. Utilizing IVLEs has minimized the dependence on dextrose as a major source of nonprotein calories and prevents the clinical consequences of essential fatty acid deficiency (EFAD). The objective of this article is to review the history of IVLE, their composition, the different generations of widely available IVLE, the variables to consider in adult patients when selecting lipids, the complications of IVLE and how to minimize them.

## 2. History of IVLE in Parenteral Nutrition

Trials of parenteral lipids for nutritional support date back to the eighteenth century. In 1712, William Courten infused olive oil into the veins of a dog that died within hours of infusion with symptoms of respiratory distress probably secondary to a fat embolism (as Courten speculated) [7,8,9]. In Canada in 1873, Edward Hodder infused fat in the form of milk into three cholera patients, two of which recovered completely, while the third patient did not survive [8,9,10]. Several others found that milk infusion led to adverse effects, which supported the earlier observation of Courten that unmodified fat, could not be given IV [10].

Between 1920 and 1960, scientists in both the United States and Japan developed and tested hundreds of fat emulsions of varying compositions [8,9]. These studies led to development of the first IVLE in the United States (Lipomul, produced by Upjohn Co. Kalamazoo, MI, USA). However, the severe and multiple complications that arose, attributed to the choice of the emulsifier, led to its withdrawal from the market and discouraged the use of IVLE in U.S. and other parts of the world that time [8,11]. Using egg phospholipid as an emulsifier together with SOY, Arvid Wretlind (commonly recognized as the “father of complete parenteral nutrition”) together with O. Schuberth introduced successfully the first nontoxic, readily available IVLE, Intralipid, in 1961 [8,11,12]. This was subsequently used in UK, France, and Scandinavia, but not in the US at that time. Concurrently, in 1968, the glucose system was introduced by Wilmore and Dudrick [13,14] that was based on administering high dose of glucose, amino acids and other nutrients without fat because IVLEs were not available at that time in the United States. On the other hand, the Swedish regimen, which provided calories from both a lipid emulsion and glucose, was referred to as the fat system [8,11]. Further study by Meguid et al. [15] using IVLE (Liposyn, 10% safflower oil-based IVLE) led to gradual return of the IVLE use as a part of PN to the US clinical practice [13]. The complications of excessive intake of parenteral dextrose as the sole energy source were increasingly identified (e.g., hepatic steatosis, respiratory insufficiency, hyperglycemia-related immunosuppression), resulting in increased acceptance for the use of the SOY-based IVLE as a daily energy source [2].

In Europe in 1984, a second generation IVLE was introduced consisting of 50:50 physical mixtures of SOY and medium chain triglycerides (MCT) that was derived from the assumption that the newly recognized SOY-related complications (i.e., reticulo-endothelial system dysfunction exaggerated systemic inflammatory response in the critically ill, and liver dysfunction in acutely ill infants and in patients of any age requiring long-term PN) [16,17,18,19,20] were possibly attributed to its high ω-6 FA content [2]. A third generation IVLE was introduced in 1990 that consisted of 20% SOY and 80% olive oil. Later that decade, an ω-3 rich Fish-oil (FO) -based IVLE constituted the fourth-generation IVLE [2]. The IVLE formulations have been classified into generations based on an American Society of Parenteral and Enteral Nutrition position paper [21,22]. The generations have primarily been defined based on the FA derivative of the IVLE, however the generations are further categorized by the inflammatory response generated by infusion of the lipid. First-generation products are typically pro-inflammatory, second and third generation products are inflammatory neutral, and fourth generation products are anti-inflammatory [21,22]. See Table 1 for composition of available parenteral oils.

## 3. Composition of IVLEs by Generation

IVLEs are unique in their FA profile and additive content (see Table 2). Thus, they may have differing biological and clinical consequences. Emerging literature has indicated that there are many benefits with utilizing alternative lipids such as olive/soy-based formulations, and combination lipids such as soy/MCT/olive/fish oil, compared with SOY based lipids, as they have less inflammatory properties, are immune modulating, have higher antioxidant content, decrease risk of cholestasis, and improve clinical outcomes of certain subgroups of patients, and have a lower association of morbidity and mortality in certain groups of patients [2]. The primary metabolites of ω-3 fatty acids are EPA and DHA, which act as precursors for anti-inflammatory factors in the body [23,24]. The precursors for the pro-inflammatory mediators (2-series prostaglandins and thromboxanes, and 4-series leukotrienes) are metabolites of ω-6 fatty acids. The ω-6:ω-3 ratio has therefore been suggested as an important factor when considering an IVLE. This has also lead to the development of various combinations of fats in IV lipids that may play a role in immunity and inflammation thus influencing patient outcomes [23,24].

SOY-based lipids have been extensively used fairly successfully for decades and are the prototype of first generation lipids. SOY-based lipids have high concentrations of PUFAs with a quarter of FAs comprised of ω-9 FAs (oleic acid), are rich in phytosterols. The ratio of ω-6: ω-3 is elevated in soybean oil, estimated at 7:1 [3]. SOY-based lipids increase the risk of intrahepatic biliary cholestasis, and the risk of sepsis by altering the migratory and phagocytic function of neutrophils in a dose and rate-dependent fashion as shown in animal models [2,25]. In addition, emulsions with a high content of ω-6 FAs have been linked with greater immunosuppressive effects [26].

MCT based lipid emulsions were developed as second generation IVLEs and included 50/50 blend of SOY and MCTs derived from coconut oils and other tropical nut oils. A blended product was required as MCTs have minimal EFAs and are unsuitable for use as a sole source of fat. MCTs are resistant to peroxidation, have less pro-inflammatory effects and easily metabolized rendering a biologically favourable profile [2]. When used in high doses, MCT oils may result in acidemia.

Third generation IVLEs are composed of an 80:20 mix between OO and SOY respectively [21,22]. The fat content of the commercially available third generation IVLE in Canada and Europe consists of 15% SFAs, 65% MUFA, and 20% essential PUFAs. MUFAs are less prone to peroxidation than PUFAs, and little to no effect on inflammatory parameters when compared with soybean or combination soybean-MCT emulsions in several studies [27]. This IVLE has been available in Canada for the past three years and in Europe for the preceding 16 years.

Fourth generation IVLEs consist of any formulation containing FO. IVLEs containing FO are the only products available that provide a large content of EPA and DHA, allowing a lower ω-6/ω-3 1:8, which is a significant departure from the ω-6: ω-3 ratios associated with the lipid emulsions of the previous generations speculated to provide an anti-inflammatory effect [22]. Omegaven^®^ (Fresenius Kabi, Bad Homberg, Germany) is the only commercially available product available in Canada, Europe and Asia marketed as 100% FO. It is a therapeutic lipid used primarily to manage PN associated liver disease (PNALD), however it is an incomplete lipid source, and when used in isolation may lead to the development of EFAD. Additionally, FO IVELE is significantly more costly than other IVLEs, and as such it is not widely used, reserved for the management of PNALD. Greater discussion on the role for FO is discussed later in this manuscript. SMOFlipid (Fresenius Kabi Limited, Cheshire, UK) is a FA combination product that contains a mix of the major FAs discussed to date. The concentrations of SOY:MCT:OO:FO respectively are 30%, 30%, 25%, and 15%. The ω-6:ω-3 ratio is 2.5:1, which is a favourable profile compared with other ω-6 rich products [21].

## 4. Clinical Implications for IVLE Selection: Selected Patient Populations

### Critical Care Patients

Despite biological plausibility and animal based studies supporting the anti-inflammatory effects of the later generation IVLEs, few clinical studies have been published on the clinical efficacy of these products. Manzanares and colleagues [28] published a systematic review and meta-analysis reviewing the role of parenteral fish oil lipid emulsions in the critically-ill population. The authors hypothesized that parenteral fish oil containing emulsions may improve clinical outcomes in the critically-ill, with conclusions that FO containing emulsions may be associated with a tendency to reduce mortality and ventilation days in the critically-ill, although this did not reach significance. No changes on infectious complications or ICU length of stay (LOS) were observed. Another meta-analysis published by Palmer and colleagues investigated the impact of ω-3 FA supplemented PN in critically-ill adults concluded that parenteral FO did not improve mortality, infectious compilations, and ICU LOS compared with standard PN [29]. Despite these lackluster findings, the current Society of Critical Care Medicine and ASPEN recommend holding or limiting SOY-based IVLE during the first week of PN therapy [30,31]. It is important to note, however, that this recommendation did not receive a consensus vote among the authors of these guidelines, and more recent studies are challenging the concept that soybean oil lipid emulsion increases the risk for infectious complications. These findings were limited to a select group of patients who were critically ill and may not be generalizable to a broader group of patients requiring PN.

## 5. Parenteral Nutrition Associated Liver Disease 

PN associated liver disease (PNALD) is an established complication in patients receiving long term PN. Liver biochemical abnormalities are often the first sign of PNALD. While most hepatic biochemical abnormalities that develop during PN administration are benign, and asymptomatic, with reversible transaminitis and hyperbilirubinemia following discontinuation of PN [2], in patients dependent on long-term PN, advanced PNALD with resultant cirrhosis and decompensated liver disease is an infrequent but fatal complication.

The relationship between lipids and the development of PNALD has been the subject of discussion in the literature since the inception of PN, although it is important to recognize that factors apart from lipid related characteristics, such as overfeeding particularly with dextrose, may be associated with the development of PNALD Additional, possible contributors to the development of PNALD include, hepatic inflammation due to bacteremia or a cholestatic cause from inadequate enterohepatic circulation of bile salts [32]. Omega-6 polyunsaturated fatty acids and the associated phytosterols in lipid emulsions have been thought to contribute to the development of hepatotoxicity, although Ng and colleagues [33] have recently shown that the addition of phytosterols to FO IVLE did not increase biomarkers of PNALD [34]. Fish oil emulsions are composed mainly of omega-3 polyunsaturated fatty acids with minimal phytosterols, and have been shown to be hepatoprotective [34]. Since lipid exposure and sepsis have been shown to be the dominant risk factors for development of PNALD, prevention of PNALD and PN prescribing practices have targeted lipid-sparing or lipid reduction strategies.

Much of the established literature assessing the impact of newer generation IVLEs on the course of PNALD are in the pediatric population. A recent systematic review and meta-analysis concluded that FO IVLE was useful in the treatment of cholestasis in pediatrics [35]. Premkumar reported outcomes in 57 infants treated for PNALD with FO IVLE between 2007 and 2011. Cholestasis resolved with fish oil therapy in over 80% of infants [36]. More recently Nandivada reported the long-term effects of FO monotherapy in infants requiring at least three years of PN, and did not demonstrate the development of EFAD [37]. However, Nandivada and colleagues did not report fatty acid profiles for long term fish oil recipients, and patients receiving FO IVLE are at risk for deficiencies in specific fatty acids despite normal triene:tetrane ratios [38].

RCTs comparing the efficacy of newer generation IVLEs to SOY in adult patients with hepatic cholestasis are lacking, although a recent study describes proof of concept evidence in which 100% FO IVLE restored liver enzymes, markers of fibrosis inflammatory cytokines to healthy values [39]. To the authors’ knowledge, only a few case reports have been published regarding the role of ω-3 FA based IVLEs in the treatment of adult patients with PNALD and support a benefit [32,40]. There is a strong biologic rationale, and emerging clinical evidence for the efficacy of FO IVLEs in mitigating the progression of PNALD. In Canada, FO IVLE remains available for compassionate use only, although SMOFlipid has been approved. Additional measures to manage PNALD may be considered alongside lipid selection, however, the management of PNALD lies beyond the scope of this article.

### Infection

Multiple mechanisms increase the risk of infectious complications in patients receiving parenteral nutrition. Compounding of parenteral nutrition, central venous catheter care and the PN prescription itself all may contribute to catheter related blood stream infections (CRBSI). Of all potential causes, the role of that IV lipid emulsions contribute to infection remains controversial. Several theories or proposed mechanisms exist to explain the rationale behind implicating IV lipids in increasing CRBSI. IV lipid emulsions have a slightly alkaline pH and are isotonic providing an optimal growth medium for microbes [41]. It has been postulated that the lipid dose and administration rate may compromise the reticuloendothelial system function leading to competition between lipid and microbes for clearance, with an end result of long chain triglyceride deposition in the liver [41]. The result is a decreased ability of Kupffer cells to free hepatic toxins, including harmful microorganisms. Finally, IV lipids may interfere with macrophage and neutrophil function [41,42]. The clinical data regarding novel IVLEs and infectious outcomes are limited and mixed. A 2012 study by Pontes-Arruda et al. [43] found no difference in CRBSI rates between patients on an olive oil based IV lipid versus those on an MCT/LCT based IV lipid. However, Grau-Carmona et al. showed a reduction in nosocomial infections in critically ill patients receiving ω-3 based lipid emulsions [44] in a critically ill population. The general consensus from various nutrition support societies is that further randomized controlled trials are required to better elucidate the role of IV lipids in CRBSIs. There is agreement that decreasing the risk of CRBSI in parenteral nutrition requires mitigating all potential risks, of which IV lipid emulsions may play a small role.

## 6. Hypertriglyceridemia and Hypercholesterolemia

Hyperlipidemia including hypertriglyceridemia is an established complication associated with some parenterally fed patients. In a healthy individual, lipid particles are hydrolyzed to release fatty acids via the action of lipoprotein lipase (LPL). These fatty acids are eventually used for energy, stored in adipose tissue, or metabolized by the liver. In a stressed patient however the activity of LPL is decreased leading to an accumulation of lipids in the blood [45]. The resultant hyperlipidemia may potentially lead to altered pulmonary gas exchange, impaired immune response, and in rare cases, acute pancreatitis [45,46]. For the latter to occur, serum triglyceride levels should exceed 1000 mg/dL (11.3 mmol/L) [46].

When monitoring serum triglyceride (TG) levels it is important to differentiate between hypertriglyceridemia associated with dextrose overfeeding or impaired lipid clearance. Other factors that predispose parenterally fed patients to elevated serum triglycerides must also be investigated. These include but are not exclusive to sepsis, multi-organ failure, obesity, diabetes, renal failure, HIV, as well as pre-existing pancreatitis and hyperlipidemia. All these conditions can affect lipid clearance, regardless of lipid source. Various medications can also potentially alter fat metabolism and contribute to hypertriglyceridemia. ASPEN guidelines suggest the risk for adverse effects are increased when the serum TG level exceeds 400 mg/dL (4.5 mmol/L) [46]. Various studies suggest this change should start with a lowering of dextrose followed by a reduction in lipid provision if serum TG levels do not normalize within a week. In cases of serum TGs exceeding 1000 mg/dL (11.3 mmol/L) IV lipid emulsions should be withheld until normalization of levels [45,46].

Additional consideration lies with fat source in the IV lipid emulsion. MCTs are oxidized faster than LCTs therefore may be cleared at a faster rate. The introduction of fish oil into IV lipid emulsions may conceivably result in TG improvement. A 2016 review of ω-3 FA in PN by Klek summarizes evidence on the effect of oral fish oil supplementation on normalization of serum lipid concentrations [23]. Further study of a dose dependent effect for PN IV lipid emulsions containing fish oil is likely warranted.

Various guidelines exist for dosing of lipids to reduce the risk of developing hyperlipidemia/hypertriglyceridemia in parenterally fed patients. For soy based lipid emulsions doses of ≤1 gram lipid/kg ideal body weight, or no more than 30% of total calories is recommended. For lipid emulsions containing a combination of oils, recommendations up to 1.7 g lipid/Kg, ideal body weight are considered safe [2]. Lipid rate must also be considered, especially with soy based emulsions. A maximum of 0.11 grams/Kg/h or 1 Kcal/Kg/h should be infused [47]. Overall, it is important to monitor TG levels weekly when initiating IV lipids, dose appropriately to maintain normal lipid levels while preventing essential fatty acid deficiency and providing a mixed substrate of energy.

### Essential Fatty Acid Deficiency

A primary indication for the use of IVLEs in patients receiving parenteral nutrition is to provide EFAs. EFAD results from insufficient quantities of linoleic acid and α-linolenic acid. Humans lack the desaturase enzyme needed to produce the ω-3 (double bond between carbons 3 and 4) and ω-6 (double bond between carbons 6 and 7) FAs. Linoleic acid (LA) (C18:2, ω-6) and alpha-linolenic acid (ALA) (C18:3, ω-3) should therefore constitute at least 2% and 0.5%, respectively, of the daily caloric intake to prevent essential FA deficiency (EFAD). Alternatively, ESPEN guidelines recommend a minimum of 7–10 grams of EFA/day. Inadequate lipid provision while on PN can lead to EFAD in two–four weeks dependent upon the patient’s underlying nutritional status. For example, 30% of patients may develop evidence of EFAD after one week, 66% at two weeks, 83% at three weeks, and 100% at four weeks in the presence of a hypercaloric, fat-free continuous PN [48]. In children, the time frame for developing EFAD may be much quicker compared with adults. Before the introduction of parenteral nutrition and the intravenous lipid emulsions, EFAD had previously been documented only in infants and appeared as a scaly rash with specific changes in the plasma FA profile [7,49,50]. Adults were thought not to be susceptible to EFAD because of sufficient FA stores in adipose tissue. However, it is now widely recognized that abnormal FA profiles in addition to clinical manifestations of EFAD are known to occur in adults with severe short bowel syndrome who are on long term total parenteral nutrition that lacks parenteral lipids [1,22,49,50,51]. Manifestations of EFAD include alterations in platelet function, abnormal liver enzymes, depressive symptoms, poor wound healing, hair loss, and dermatitis. The development of clinical signs of deficiency is usually preceded by elevated liver enzymes.

Novel subsequent generation intravenous lipid emulsions have varying proportions of essential fatty acids as compared to soybean oil emulsions [52]. Although the disadvantages of soybean oil lipid emulsions are highlighted in the PNALD discussion, the advantage of soybean oil emulsions is the high content of omega-6 fatty acids required to prevent EFAD. With alternative lipid emulsions becoming more prevalent across North America, the potential of developing EFAD may increase (see Table 3 for the essential fatty acid content in selected IVLEs). In particular, patients receiving FO IVLE may be at increased risk for deficiencies in specific fatty acids despite normal triene:tetrane ratios.

With the development of EFAD, one of the first biochemical signals is the accumulation of Mead acid and a decrease in linoleic acid. Oleic acid (predominant in olive oil) is the precursor to Mead acid. An elevated Mead acid and reduced linoleic acid and arachidonic acid level, which is manifested as an elevated triene/tetraene ratio of Mead acid/arachidonic acid confirms a diagnosis of EFAD [52]. In patients receiving novel IVLE, an elevated triene/tetraene ratio may simply be reflective of the composition of the lipid emulsion, and does not necessarily indicate the development of EFAD. In North America, lipids were historically high in in tetraenes (linoleic acid and ALA from soybean oil) and low in trienes (olive oils). The literature definition of EFAD is a triene/tetraene ratio > 0.2, and other proposed definitions may suggest a lower ratio may be more reflective of fatty acid profiles [52].

## 7. Contraindications to IVLEs

Olive-oil based IVLEs are contraindicated in individuals with known hypersensitivity to egg, olive and soybean, peanut proteins, or to any active ingredient, while SMOFlipid should be avoided in patients who have hypersensitivity to fish, egg, soybean, or peanut protein. Allergy contraindications with standard soybean IVLEs include hypersensitivities to egg, soy, or peanut proteins. IVLE drug monographs caution about potential cross contamination for individuals with peanut protein allergy in soy allergic individuals [53].

Generally, IVLEs should be avoided or used with caution in the presence of severe hyperlipidemia (including severe disorders of lipid metabolism characterized by hypertriglyceridemia-associated acute pancreatitis), critically ill adults with shock, recent cardiac infarction, stroke, embolism, undefined coma status and severe liver insufficiency. OO based IVLEs should be administered with caution for patients who require anticoagulation therapy, as this lipid only contains 10–50 µg/L of vitamin K.

Soybean based IVLEs are appropriate to consider for individuals who have or are at risk of essential fatty acid deficiency and those patients who are allergic to fish or olive oil [1,4].

## 8. Special Considerations in Lipid Selection

General recommendations are to dose IVLE no greater than 1 g/kg body weight/day and to not exceed 2.5 g/kg body weight per day [1]. Maximum doses for IVLEs are 2.5 g/kg body/day for soy-based IVLE, 2 g/kg body weight/day for olive-oil based IVLE, and 2 g/kg bodyweight/day for fish-based IVLE [53]. Fat overload syndrome is caused by the impaired ability to eliminate triglycerides [34]. This syndrome can happen from excessive infusion rates of any IVLE greater than 0.08–0.13 g/kg/h [53]. It is recommended to stop fish-based IVLE in situations when a patient’s triglyceride level is greater than 3 mmol/L (265 mg/dL). In contrast, soy-based and olive-oil based IVLE should be reduced or discontinued if a patient’s triglyceride exceeds 4.5 [53]. There are no specific guidelines to assist a clinician to reinstate IVLEs once discontinued in this context, however, it would be reasonable to allow the triglyceride level normalize, followed by cautious lipid addition with vigilant biochemical monitoring.

In pregnancy, PN chould be considered if the pregnant patient has failed attempts with enteral nutrition and/or malabsorptive disorders and established degree of weight loss over a period of at least four weeks, with electrolyte abnormalities, along with progressive hypoalbuminemia. Case reports and case series have demonstrated the safety and efficacy of IVLE use for Home PN during pregnancy and hyperemesis gravidarum. Most of the experience with IVLE in pregnancy has been with SOY.

## 9. Conclusions

Biological rationale and experimental studies have paved the way toward considerable advances in the development of IVLEs over the past two decades, with subsequent generations entering the global market. Access to IVLEs is variable, ranging from a full complement of IVLEs available in parts of Europe to SOY predominance in the United States. Early signals have been observed in clinical studies for benefits of later generation IVLEs on health outcomes. However, the literature is still limited, but is growing. Data are consistently favourable for FO IVLE in the treatment of pediatric and neonatal patients with PNALD, while data in adult patients is limited to mainly case reports with encouraging findings. Collaborations between hospital pharmacies and nutrition services need to be developed to ensure best lipid selection, recognizing the impact that availability affects choice. Ultimately, the objectives of IVLE selection should revolve around optimizing clinical benefits, dosing, and administration to reduce harm imposed by IVLE choice.

## Figures and Tables

**Table 1:** Oils in intravenous lipid emulsions (modified from Fell et al. [3]).

Lipid Component	Soybean	Safflower	Olive	Fish	Coconut
FA composition (%)					
LA (ω-6)	50	77	4	1–3	2
ARA (ω-6)	0	0	0	0	0
Alpha-ALA (ω-3)	10	0	0	1.3–5.2	0
EPA (ω-3)	0	0	0	5.4–13.9	0
DHA (ω-3)	0	0	0	5.4–26.8	0
Oleic acid (ω-9)	25	15	85	16–20	6
MCT	0	0	0	0	65
SFAs	15	8	11	10–20	27
Phytosterols conc. (mg/100 mg oil)	300	450	200	Trace	70
Alpha-Tocopherol conc. (mg/100 mg oil)	6.4–7.5	34	10–37	45–70	0.2–2

LA: Linoleic acid, ARA: Arachidonic acid, Alpha-ALA: Alpha-Linolenic acid, EPA: eicosapentaenoic acid, DHA: docosahexaenoic acid, MCT: Medium chain triglycerides, SFAs: Saturated fatty acids.

**Table 2:** Selected commercially available intravenous fat emulsion products.

Product Name	Lipid Source	Linoleic (%)	α-Linolenic (%)	ω-6: ω-3 Ratio	α-Tocopherol, mg/L	Phytosterols, mg/L
Intralipid^®^ 10%, 20%, 30%	100% soybean oil	44–62	4–11	7:1	38	348 ± 33
Structolipid^®^ 20%	64% soybean oil 36% MCT	35	5	7:1	6.9	NA
Lipofundin^®^ MCT/LCT 10%, 20%	50% soybean oil 50% MCT oil	27	4	7:1	85 ± 20	NA
ClinOleic^®^ 20%	20% soybean oil 80% olive oil	18.5	2	9:1	32	327 ± 8
SMOFlipid^®^ 20%	30% soybean oil, 30% MCT, 25% olive oil, 15% fish oil	21.4	2.5	2.5:1	200	47.6
Omegaven^®^ 10%	100% fish oil	4.4	1.8	1:8	150–296	0

MCT: medium-chain triglyceride, ω-6: ω-3 Ratio: ratio of ω-6 fatty acids to ω-3 fatty acids, NA: not available. A Adapted from [3,6,14].

**Table 3:** Essential fatty acid doses in selected intravenous lipid emulsions (IVLEs).

Lipid Emulsion	Lipid Source	g EFA/mL of Ivle	Estimate of Daily mL of Ivle Required/1000 kcals to Prevent EFAD * (4% of Total Energy)
Intralipid 20%	100% soybean oil	0.12	38
ClinOleic 20%	20% soybean oil/80% olive oil	0.04	113
SMOFlipid 20%	30% soybean oil/30% MCT oil/25% olive oil/15% fish oil	0.04 (mean)	113
Propofol 10%	100% soybean oil	0.06	75

* If hypocaloric parenteral nutrition (PN) delivered in obese patient, ml required to prevent essential fatty acid deficiency (EFAD) will be less than quoted.

## References

[1] Carpentier Y.A., Dupont I.E. (2000). Advances in intravenous lipid emulsions. World J. Surg..

[2] Anez-Bustillos L., Dao D.T. (2016). Intravenous Fat Emulsion Formulations for the Adult and Pediatric Patient: Understanding the Differences. Nutr. Clin. Pract..

[3] Fell G.L., Nandivada P. (2015). Intravenous Lipid Emulsions in Parenteral Nutrition. Adv. Nutr..

[4] Waitzberg D.L., Torrinhas R.S. (2006). New parenteral lipid emulsions for clinical use. J. Parenter. Enter. Nutr..

[5] Driscoll D.F. (2006). Lipid injectable emulsions. Nutr. Clin. Pract..

[6] El Kasmi K.C., Anderson A.L. (2013). Phytosterols promote liver injury and Kupffer cell activation in parenteral nutrition-associated liver disease. Sci. Transl. Med..

[7] Lee E.J., Simmer K. (1993). Essential fatty acid deficiency in parenterally fed preterm infants. J. Paediatr. Child Health.

[8] Jeppesen P., Hoy C.-E. (1998). Essential fatty acid deficiency in patients receiving home parenteral nutrition. Am. J. Clin. Nutr..

[9] Bistrian B.R. (2003). Clinical aspects of essential fatty acid metabolism: Jonathan Rhoads Lecture. J. Parenter. Enter. Nutr..

[10] Das U.N. (2006). Essential fatty acids: Biochemistry, physiology and pathology. Biotechnol. J..

[11] Le H.D., Meisel J.A. (2009). The essentiality of arachidonic acid and docosahexaenoic acid. Prostaglandins Leukot. Essent. Fatty Acids.

[12] Courten W., Sloane H. (1710). Experiments and observations of the effects of several sorts of poisons upon animals, etc. made at Montpellier in the years 1678 and 1679, by the late William Courten Esq; communicated by Dr. Hans Sloane, R.S. Secr. Translated from the Latin MS. Philos. Trans..

[13] Vinnars E., Wilmore D. (2003). Jonathan Roads Symposium Papers. History of parenteral nutrition. J. Parenter. Enter. Nutr..

[14] Vassilyadi F., Panteliadou A.K. (2013). Hallmarks in the history of enteral and parenteral nutrition: From antiquity to the 20th century. Nutr. Clin. Pract..

[15] Hodder E. (1873). Transfusion of milk in cholera. Practitioner.

[16] Mundi M.S., Salonen B.R. (2016). Home Parenteral Nutrition: Fat Emulsions and Potential Complications. Nutr. Clin. Pract..

[17] Schuberth O., Wretlind A. (1961). Intravenous infusion of fat emulsions, phosphatides and emulsifying agents. Acta Chir. Scand. Suppl..

[18] Wilmore D.W., Dudrick S.J. (1968). Growth and development of an infant receiving all nutrients exclusively by vein. JAMA.

[19] Dudrick S.J., Macfadyen B.V. (1972). Parenteral hyperalimentation: Metabolic problems and solutions. Ann. Surg..

[20] Meguid M.M., Schimmel E. (1982). Reduced metabolic complications in total parenteral nutrition: Pilot study using fat to replace one-third of glucose calories. J. Parenter. Enter. Nutr..

[21] Biesboer A.N., Stoehr N.A. (2016). A Product review of alternative oil-based intravenous fat emulsions. Nutr. Clin. Pract..

[22] Vanek V.W., Seidner D.L. (2012). A.S.P.E.N. position paper: Clinical role for alternative intravenous fat emulsions. American Society for Parenteral and Enteral Nutrition (A.S.P.E.N.) Board of Directors. Nutr. Clin. Pract..

[23] Klek S. (2016). Omega-3 Fatty Acids in Modern Parenteral Nutrition: A Review of the Current Evidence. J. Clin. Med..

[24] Miles E., Calder P. (2015). Fatty Acids, Lipid Emulsion and the Immune and Inflammatory Systems. World Rev. Nutr. Diet..

[25] Clayton P.T., Whitfield P. (1998). The role of phytosterols in the pathogenesis of liver complications of pediatric parenteral nutrition. Nutrition.

[26] Calder P.C., Yaqoob P. (1987). Fatty acids and lymphocyte functions. Br. J. Nutr..

[27] Reimund J.M. (2005). Efficacy and Safety of an Olive Oil-based Intravenous Fat Emulsion in Adult Patients on Home PN. Aliment. Pharmacol. Ther..

[28] Manzaneres W., Dhaliwal R. (2014). Parenteral fish oil lipid emulsions in the critically ill: A systematic review and meta-analysis. J. Parenter. Enter..

[29] Palmer A.J., Ho C.K. (2013). The role of w-3 fatty acid supplemented parenteral nutrition in critical illness in adults: A systematic review and meta-analysis. Crit. Care Med..

[30] Taylor B.E., McClave S.A. (2016). Guidelines for the provision and assessment of nutrition support therapy in the adult critically ill patient: Society of Critical Care Medicine (SCCM) and American Society for Parenteral and Enteral Nutrition (A.S.P.E.N.). Crit. Care Med..

[31] Critical Care Nutrition Canadian Clinical Practice Guidelines, Composition of Parenteral Nutrition: Type of Lipids 2013. www.criticalcarenutrition.com.

[32] Burns D.L., Gill B.M. (2013). Reversal of parenteral nutrition-associated liver disease with a fish oil-based lipid emulsion (Omegaven) in an adult dependent on home parenteral nutrition. J. Parenter. Enter..

[33] Ng K., Stoll B. (2016). Vitamin E in new generation lipid emulsions protects against parenteral nutrition-asociated liver disease in parenteral nutrition-fed preterm pigs. J. Parenter. Enter.

[34] Fallon E.M., Le H.D. (2010). Prevention of parenteral nutrition-associated liver disease: Role of omega-3 fish oil. Curr. Opin. Transplant..

[35] Park H.W., Lee N.M. (2015). Parenteral fish oil containing lipid emulsions may reverse parenteral nutrition-associated cholestasis in neonates: A systematic review and meta-analysis. J. Nutr..

[36] Premkumar M.H., Carter B.A. (2013). High rates of resolution of cholestasis in parenteral nutrition-associated liver disease with fish-oil based lipid emulsion monotherapy. J. Pediatr..

[37] Nandivada P., Fell G.L. (2016). Long term fish-oil lipid emulsion use in children with intestinal failure-associated liver disease. J. Parenter. Enter..

[38] Le H.D., de Meijer V.E. (2011). Parenteral fish-oil based lipid emulsion improves fatty acid profiles and lipids in parenteral nutrition-dependent children. Am. J. Clin. Nutr..

[39] Pastor-Cleriques A., Marti-Bonmati E. (2014). Anti-inflammatory and anti-fibrotic profile of fish oil emulsions used in parenteral nutrition-associated liver disease. PLoS ONE.

[40] Xu Z., Li Y., Wang J. (2013). Effect of omega-3 polyunsaturated fatty acids to reverse biopsy-proven parenteral nutrition-associated liver disease in adults. Clin. Nutr..

[41] Cheng M.L., Heidbreder C. (2013). Infectious Complications with Nondaily versus Daily Infusion of IV Fat Emulsions in Non-Critically Ill Adults. Nutr. Clin. Pract..

[42] McCleary E.J., Tajchman S. (2016). Parenteral Nutrition and Infection Risk in the ICU: A Practical Guide for the Bedside Clinician. Nutr. Clin. Pract..

[43] Pontes-Arruda A., Dos Santos M.C. (2012). Influence of PN Delivery System on the Development of Bloodstream Related Infections in Critically Ill Patients: An International, Multicenter, Prospective, Open-Label, Controlled Study-EPICOS Study. J. Parenter. Enter..

[44] Grau-Carmona T., Bonet-Saris A. (2015). Influence of n-3 polyunsaturated fatty acids enriched lipid emulsions on nosocomial infections and clinical outcomes in critically ill patients: ICU Lipids Study. Crit. Care Med..

[45] Btaiche I., Khalidi N. (2004). Metabolic Complications of parenteral nutrition in adults, part 1.. Am. J. Health-Syst. Pharm..

[46] Mueller C. (2012). The A.S.P.E.N. Adult Nutrition Support Core Curriculum.

[47] Driscoll D. (2017). Pharmaceutical and Clinical Aspects of Lipid InjecEmulsions. J. Parenter. Enter..

[48] Goodgame J.T., Lowry S.F. (1978). Essential fatty acid in total parenteral nutrition: Time course of development and and suggestions for therapy. Surgery.

[49] Cooke R.J., Zee P. (1984). Essential fatty acid status of the premature infant during short-term fat-free parenteral nutrition. J. Pediatr. Gastroenterol. Nutr..

[50] Friedman Z., Danon A. (1976). Rapid onset of essential fatty acid deficiency in the newborn. Pediatrics.

[51] St-Onge M.P., Bosarge A. (2008). Medium chain triglyceride oil consumption as part of a weight loss diet does not lead to an adverse metabolic profile when compared to olive oil. J. Am. Coll. Nutr..

[52] Gramlich L., Meddings L. (2015). Essential fatty acid deficiency in 2015: The impact of novel intravenous lipid emulsions. J. Parenter. Enter.

[53] Gramlich L, Atkins M. (2017). Alberta Health Services Nutrition Practice Guidelines–Intravenous Lipid Emulsions.

